# LTR-Retrotransposons in *R. exoculata* and Other Crustaceans: The Outstanding Success of GalEa-Like Copia Elements

**DOI:** 10.1371/journal.pone.0057675

**Published:** 2013-03-04

**Authors:** Mathieu Piednoël, Tifenn Donnart, Caroline Esnault, Paula Graça, Dominique Higuet, Eric Bonnivard

**Affiliations:** 1 UMR 7138 Systématique Adaptation Evolution, Equipe Génétique et Evolution, Université Pierre et Marie Curie, Paris, France; 2 Systematic Botany and Mycology, University of Munich (LMU), Munich, Germany; Ben-Gurion University, Israel

## Abstract

Transposable elements are major constituents of eukaryote genomes and have a great impact on genome structure and stability. They can contribute to the genetic diversity and evolution of organisms. Knowledge of their distribution among several genomes is an essential condition to study their dynamics and to better understand their role in species evolution. LTR-retrotransposons have been reported in many diverse eukaryote species, describing a ubiquitous distribution. Given their abundance, diversity and their extended ranges in C-values, environment and life styles, crustaceans are a great taxon to investigate the genomic component of adaptation and its possible relationships with TEs. However, crustaceans have been greatly underrepresented in transposable element studies. Using both degenerate PCR and *in silico* approaches, we have identified 35 Copia and 46 Gypsy families in 15 and 18 crustacean species, respectively. In particular, we characterized several full-length elements from the shrimp *Rimicaris exoculata* that is listed as a model organism from hydrothermal vents. Phylogenic analyses show that Copia and Gypsy retrotransposons likely present two opposite dynamics within crustaceans. The Gypsy elements appear relatively frequent and diverse whereas Copia are much more homogeneous, as 29 of them belong to the single GalEa clade, and species- or lineage-dependent. Our results also support the hypothesis of the Copia retrotransposon scarcity in metazoans compared to Gypsy elements. In such a context, the GalEa-like elements present an outstanding wide distribution among eukaryotes, from fishes to red algae, and can be even highly predominant within a large taxon, such as Malacostraca. Their distribution among crustaceans suggests a dynamics that follows a “domino days spreading” branching process in which successive amplifications may interact positively.

## Introduction

Transposable elements (TEs) have a large impact on genome structure and stability, and are therefore considered as one of the major sources of genetic variability in eukaryotes [1]–[4]. Environmental variations can promote genome plasticity through transcriptional activation and TE mobilization, often in response to specific stimuli such as biotic stress (e.g., pathogens) and abiotic environmental changes [5]–[9]. Retrotransposons, a TE class specific to eukaryotes, transpose *via* a RNA intermediate. Five orders of retrotransposons can be defined based on their structural features and their phylogenetic relationships [10]: Long Terminal Repeat retrotransposons (LTR-retrotransposons), tyrosine recombinase encoding retrotransposons (e.g. DIRS1-like elements), Penelope elements, LINEs (Long INterspersed Elements) and SINEs (Short INterspersed Elements). Copia (or Ty1/Copia), Gypsy (or Ty3/Gypsy) and BEL/Pao elements constitute the three superfamilies of LTR-retrotransposons. These elements are related to retroviruses [11] and usually encode two Open Reading Frames (ORFs). The first ORF, the *gag* region, encodes proteins that form the virus-like particles. The second ORF, the *pol* region, is a polyprotein comprising the different domains involved in the retrotransposition mechanism. These domains include an aspartic protease (PR), a reverse transcriptase (RT), a RNase H (RH) and a DDE-type integrase (INT), whose order varies among LTR-retrotransposon superfamilies [12].

Transposable elements have been found in all eukaryotic species investigated so far [10]. However, the TE superfamilies show variable distributions among eukaryotes. For example, LINEs, SINEs retrotransposons and the Tc1/Mariner transposons, have been detected almost ubiquitously [10], [13], [14]. The Penelope retrotransposons are widely distributed among animal species, but seem to be rare among plants, protists and fungi [15]. The DIRS1-like elements are less frequent but their distribution appears broader than it was previously thought, especially in unikont species, although they remain undetectable in mammals [16]. Now reported in 61 species, they are widely distributed in some particular phyla such as Decapoda [17]. Finally, LTR-retrotransposons are found in a wide continuous range of species [18], [19], [20], but a recent analysis of 62 sequenced metazoan genomes underlined their uneven relative abundances among these species [21]. Gypsy elements are the most abundant, the BEL/Pao elements appear intermediate and the Copia retrotransposons constitute a distant third group of low-copy elements.

The decapods (shrimps, lobsters, crabs, etc), and more globally the crustaceans, are a great model to investigate the genomic component of adaptation and its possible relationship with TEs. First, crustaceans form a very large group of arthropods that exhibit great diversity in terms of species, lifestyles (including some parasitic organisms such as *Sacculina carcini*) and are found in various environments (e.g. from fresh to highly salty water or from deep-sea vents to terrestrial species). Second, they exhibit great variations in genome size; decapods range from 1.05 Gb in the crab *Carcinus maenas* to 40 Gb in the shrimp *Sclerocrangon ferox* [22], with several species (e.g. shrimps) that show particularly large genomes and are thus likely to harbor high TE contents [23]. Most of the previous studies on TEs focused on model organisms, such as studies on horizontal transfer across Mammals [24], LINEs and SINEs in human genome [25] or dynamics and impact of TE invasion on the Drosophila genomes [26]. This species sampling bias could potentially affect our knowledge in TE dynamics and evolution. This is particularly striking for marine species such as crustaceans. Given their abundance and diversity, Crustacea and Decapoda have been greatly underrepresented in studies on retrotransposons where few elements have been described to date. LINEs are the most reported retrotransposons in crustaceans with several elements described in the isopod *Porcellio scaber* [27], the ostracod *Darwinula stevensoni* [28], the branchiopod *Daphnia pulex* [29] and several decapods, principally the prawns *Litopenaeus stylirostris*, *Litopenaeus vannamei* and *Penaeus monodon* [30], [31]. DIRS1-like elements also constitute a well-studied retrotransposon group within crustaceans. They appear widely distributed among decapods with elements described in 15 diverse species [17]. Interestingly, the study of these elements revealed that they constitute a new DIRS1-like clade, called AlDIRS1 and distant from the elements identified in the *D. pulex* genome [17], [32]. This suggests that different TE dynamics occurred among the crustacean orders. By contrast, only a little is known about Penelope elements and LTR-retrotransposon distributions in crustaceans. Penelope elements have been reported only in the prawns *P. monodon* [33] and *Marsupenaeus japonicus* [34]. LTR-retrotransposons are limited essentially to those described in the sequenced genome of *D. pulex* [32] and in galatheid squat lobsters [35], [36]. Copia elements, discovered in galatheids using degenerate PCR, define the new GalEa clade, which is widely dispersed among animal species. Indeed, the GalEa-like elements have also been described in phylogenetically distant species, the teleosts *Danio rerio* (Zeco1) and *Oryzias latipes* (Olco1), and the urochordate *Ciona intestinalis* (Cico1) [35].

In this study, we particularly focus on *Rimicaris exoculata*. This deep-sea vent organism may present particular TE characteristics due to its peculiar adaptive abilities and its relatively large genome (10.16 Gb; [17], [23]). Deep-sea vents are chemosynthetic environments particularly unstable, where intense physico-chemical shifts are occurring over very short spatial and temporal scales [37]–[39]. Such unstable environment may be difficult to live in, therefore hydrothermal ecosystems are often considered harsh and stressful. They show however a much higher density of individuals compared with surrounding abyssal plains. *R. exoculata* represents an emblematic species of the Mid-Atlantic Ridge, where populations can reach up to 2500 individuals per square meter [40], and is exceptional among crustaceans for its association with bacteria [41]. It usually lives between 15°C and 30°C, but can endure sudden changes of thermal conditions due to fluid convections and survive the exposure to very high temperature vent emissions [42], [43].

While studying DIRS1-like retrotransposons in decapods, we recently characterized RexAlvi1 and RexAlvi2, two elements from *R. exoculata* [17]. Herein we characterized Copia and Gypsy retrotransposons in this species using PCR strategies, and we determined the diversity of these elements among crustaceans using both PCR and *in silico* approaches. We studied 26 species that allow us a broad coverage of the crustacean diversity. We focused in particular on 20 decapods (including 7 other hydrothermal species) that represent the major Decapoda infraorders.

## Materials and Methods

### Biological Materials

One specimen of *R. exoculata* and one specimen of each shrimps *Alvinocaris markensis*, *Mirocaris fortunata* and *Chorocaris chacei* come from the Mid-Atlantic Ridge vent fields Rainbow and were sampled with the suction sampler of the ROV (Remotely Operated Vehicle) ‘Victor 6000’ operating from the R/V “Pourquoi pas?” (cruise MoMARETO [44], August 2006, IFREMER). The second specimen of *R. exoculata* was sampled on the same field using the French “Nautile” deep-submergence vehicle operating from the R/V “Pourquoi pas?” (cruise MoMARDREAM-naut [45], July 2007, IFREMER). One specimen of each other hydrothermal decapods were collected using the French “Nautile” deep-submergence vehicle operating from the N.O. “L’Atalante”: shrimps *Alvinocaris lusca* and *Nematocarcinus burukovskyi* on the North East Pacific Rise (cruise MESCAL, June 2010, IFREMER); crab *Bythograea thermydron* and galatheid squat lobsters *Munidopsis recta* on the South East Pacific Rise (cruise BIOSPEEDO [46], March-May 2004, IFREMER). The coastal decapods (the caridean shrimps *Palaemon serratus*, *Crangon crangon* and the brachyuran crabs *Maja squinado*, *Necora puber*) and the parasitic barnacle *S. carcini* were collected in French Brittany (Roscoff, 2009). Two specimens of galatheid squat lobsters from seamounts (*Agononida laurentae* and *Eumunida annulosa*) were collected south of New-Caledonia on Norfolk seamounts during the prospecting campaigns Norfolk1 (2001, IRD Nouméa) and Norfolk 2 (2003, MUSORSTOM). The crayfish (*Orconectes limosus*) was collected near Paris (Val d'Oise) and the farmed prawns originated from Thailand (*L. vannamei*, *P. monodon*) were purchased frozen in a grocery store. Hydrothermal specimens were collected during official oceanographic research cruises; other organisms are not endangered or protected species and were not collected in privately-owned or protected areas; so, no specific permits were required for the described field studies.

For all samples, living specimens were fixed immediately after collection in liquid nitrogen for vent species, or in 70% ethanol for the other species. They were then stored at –80°C or 4°C, respectively. DNA from one individual per species was isolated from abdominal muscle tissue using the CTAB method. Dry DNA pellets were resuspended in water.

### Detection of LTR-retrotransposons Using Degenerate Primers

To isolate LTR-retrotransposon *pol* fragments, we performed PCRs using several degenerate primer pairs designed within the conserved motifs of the RT/RH domains. Three primers (GD1, GD2 and GD3) were designed to amplify motifs of Gypsy retrotransposons: ‘RMPFGL’ (5′-MGNMTGCCNTTYGGNYT-3′), ‘LTTDAS’ (5′-WSNGCRTCNGTNGSNA-3′) and ‘ADALSR’ (5'-CKNGANASNSCRTCNGC-3'). For Copia retrotransposons, we used the primer pair (CD1/CD2) that previously allowed the detection of elements in the galatheid squat lobsters [35]. CD1 corresponds to the ‘KARLVA’ motif (5′-ARRGCNMGNYTNGTNGC-3′, [35]) and CD2 to the ‘YVDD’ motif (5′-ANNANRTCRTCNACRTA-3′, [47]).

PCR amplifications were performed for 35 cycles (94°C for 45 s, 50.2°C for 1 min and 72°C for 1 min) using 50 ng of DNA, 2.5 U of Taq DNA polymerase (Promega) and 50 pmol of each degenerate primer in a final volume of 25 µL. PCR amplification products were separated on 1% agarose gels. Bands with the expected molecular weight were excised, purified with the Nucleospin Extract kit (Macherey_Nagel) and cloned in pGEM-T vector according to the manufacturer recommendations (Promega, Madison, WI, USA). One to three clones were sequenced (http://www.beckmangenomics.com) and the nucleotide sequences were submitted to the GenBank database (see Table S1 for accession numbers).

### Characterization of the Retrotransposons in *R. exoculata*

Sequences obtained with degenerate primers allowed the identification of several new LTR-retrotransposon families in *R. exoculata*. As described in Piednoël and Bonnivard [17], a group of sequences is considered as a family if its highest intra-group divergence is lower than its inter-groups divergence, without overlap of the two distributions. Two PCR walking approaches, ‘PCR walking’ [48] and ‘TE Walking’ [17], were then performed to extend large sequences from one representative initial fragment (see Table S1 for sequence reconstruction and primers). PCR amplifications were performed as presented above and for each walking step one to three clones were sequenced. Each new sequence was manually validated as an extension of the initial fragment using a minimum overlap of 50 bp between the two sequences, and a minimum DNA identity of 95%. Chimeric consensus elements were finally determined by joining the different PCR fragments using the Cap contig assembly program included in BioEdit [49].

We developed an efficient strategy that allows characterizing all parts of a full-length LTR-retrotransposon with the fewest possible PCR steps (Figure S1). (1) Detection of fragment of the RT domain using degenerate primers that can be used as an anchor sequence for PCR walking. This anchor sequence is compared with closely related retrotransposons to extrapolate the putative Primer Binding Site (PBS) sequence of the element. (2) Then the 5' edge of the element is obtained using a peculiar ‘TE walking’ step, we call ‘PBS walking’, which associates two specific primers designed within the anchor fragment and on the PBS sequence, respectively. When necessary, an additional ‘PCR walking’ step may be done to extend the 5' edge of the anchor fragment prior to the ‘PBS walking’. (3) The 5' LTR sequence is determined by ‘PCR walking’. (4) Assuming that both LTRs are almost identical, the missing 3′ part of the element is amplified using a pair of specific primers designed in the presumed 3′LTR and in the anchor fragment, respectively.

### Transcriptomic Survey

To identify transcriptionally active copies of the elements in *R. exoculata*, total RNAs were isolated from about 20 mg of abdominal muscle tissue (RNeasy mini kit, Quiagen). Prior to cDNA synthesis (Omniscript RT kit with poly(T) primer, Qiagen), RNA isolation products were treated with DNase I (10 U at 37°C during 1h30, inactivation 10 mn at 65°C). To test for DNA contamination within the RNA sample, we performed PCR amplifications using primers specific to the RT domain of each newly described element (primer sequences available upon request, see Table S1 for details). It results in an absence of PCR-amplified fragments, which attests the efficiency of the DNase treatment and the absence of the DNA contamination in the RNA sample. PCR amplifications were performed for 30 cycles (94°C for 45 sec, 54°C for 1 min, and 72°C for 1 min, followed by a final extension step at 72°C for 10 min) using about 50 ng of cDNA, 2.5 U of Taq DNA polymerase (Promega) and 10 pmol of each primer in a final volume of 25 µl.

### Data Mining

To identify Copia and Gypsy elements in various crustacean species, we screened several genomic or transcriptomic databases. Gypsy and Copia sequences from the sequenced genome of *D. pulex* were obtained either from National Center for Biotechnology Information (http://www.ncbi.nlm.nih.gov) or RepBase (http://www.girinst.org/server/RepBase/index.php). Transcriptomic sequences from Antarctic krill *Euphausia superba* [50] and those of *Euphausia crystallorophias* were kindly provided by JY Toullec (Station biologique de Roscoff); those from the amphipod *Parhyale hawaiensis* were obtained from DOE Joint Genome Institute (http://genome.jgi-psf.org/parha/parha.info.html) and those from the porcelain crab *Petrolisthes cinctipes* from Tagmount [51] (http://sequoia.ucmerced.edu/PetrolESTes/index.php). We also investigated nucleotide collection (nr/nt), expressed sequence tags (est) and whole-genome shotgun (wgs) databases from the NCBI, the Marine Genomics Project database (http://www.marinegenomics.org) and the Penaeus Genome Database (http://sysbio.iis.sinica.edu.tw/page/). Similarity searches were performed using the TBLASTX program [52]. To avoid any bias that would favor the detection of GalEa clade elements [35], two different Copia elements were used as queries: the *Drosophila melanogaster* transposable element Copia (X02599.1) and the chimeric sequence of CoRex2 (herein described). Only the *pol* sequence of GyRex2 (herein described) was used as query to detect Gypsy elements.

To investigate the distribution of GalEa-like elements in all eukaryotes, we performed TBLASTX searches on all NCBI databases using GalEa1 (DQ913005.1) and Zeco1 (DQ91300) *pol* sequence as queries. When possible, chimeric sequences of the newly identified GalEa-like elements were designed. In few cases, the sequences from one species do not overlap themselves, we were thus unable to check whether they belong to the same element or not. Subsequently, we tested the GalEa clade affiliation of the newly identified elements using two different approaches: sequences covering the RT/RH domains were included into phylogenic analyses whereas the remaining sequences were classified using similarity searches using BLAST on the Gypsy Database 2.0 [19]. In the latter case, an element was assigned to the GalEa clade under the two conditions: (i) the five best hits must correspond to the five GalEa1-like elements referenced in the database; and (ii) the difference between the best E-values obtained with GalEa-like and other reference elements must be greater than 1e-10.

### Sequence Analysis

Multiple alignments of DNA and protein sequences were constructed using MAFFT [53] and manually curated using BioEdit. Pairwise distances were estimated using the option pairwise deletion of gaps in MEGA5.0 [54] and the p-distance model. Amino acid consensus sequences of elements were constructed by identifying the most common amino acid for each position. Ambiguously aligned sites within amino acid multiple alignments were removed using BMGE [55]. Phylogenic analyses were conducted using the Neighbor Joining method [56] and the best-fit model JTT+G [57] in MEGA5.0. For all phylogenic analyses, individual clade support was evaluated by non-parametric bootstrapping [58] using 100 bootstrap replicates.

### Accession Numbers

The sequences obtained in this study have been submitted to the GenBank database (GenBank: HF548722–HF548824).

The accession numbers of the Copia elements used in phylogenetic analyses are:

*Drosophila melanogaster* 1731, X07656.1; Xanthias, FJ238509.1; *Arabidopsis thaliana* Araco, AC079131.4; Endovir1-1, AY016208.1; *Drosophila simulans* Copia, D10880.1; *Phaeodactylum tricornutum* CoDi4.4, EU432484.1; CoDi5.1, EU432486.1; CoDi6.4, EU432495.1; CoDi6.6, EU432497.1; CoDi7.1, EU432499.1; *Thalassiosira pseudonana* CoDi5.5, EU432490.1; CoDi6.1, EU432492.1; CoDi6.2, EU432493.1; *Zea mays* Hopscotch, AC084320.10; Opie-2, AC104473.2; Sto-4, AF082133.1; *Nicotiana tabacum* Tnt-1, X13777.1; Tto1, D83003.1; *Volvox carteri* Osser, X69552.1; *Oryza longistaminata* Retrofit, AH005614.1; *Saccharomyces exiguus* Tse1, AJ439547.1; *Saccharomyces cerevisiae* Ty4, M94164.1; *Vitis vinifera* Vitico1-1, AM465428.1; *Bombyx mori* Yokozuna, AB014676.1.

The accession numbers of the Gypsy elements are:

*D. melanogaster* 17.6, X01472.1; 297, X03431.1; Gypsy, M12927.1; Idefix, AJ009736.1; Springer, AF364549.1; *Tripneustes gratilla* SURL, M75723.1; *Beta vulgaris* Beetle1, AJ539424.1; *Schistosoma mansoni* Boudicca, AY662653.1; *Colletotrichum gloeosporioides* Cgret, AF264032.1 and AF264028.1; *Z. mays* Cinful-1, AF049110.1; CRM, AY129008.1; *Lycopersicon esculentum* Galadriel, AF119040.1; *A. thaliana* Gimli, AL049655.2; *Magnaporthe grisea* Grasshopper, M77661.1; MGLR3, AF314096.1; *Hydra magnipapillata* Hydra2-1, NW_002123104.1; *Pinus radiata* Ifg7, AJ004945.1; *B. mori* Kabuki, AB032718.1; Mag, X17219.1; *Musa acuminata* Monkey, AF143332.1, AF399948.1 and AF399938.1; *Drosophila buzzatii* Osvaldo, AJ133521.1; *Pisum sativum* Peabody, AF083074.1; *Alternaria alternata* Real, AB025309.1; *Oryza sativa* Retrosat-2, AF111709.1; RIRE2, AB030283.1; *Fusarium oxyporum* Skippy, L34658.1; *Strongylocentrotus purpuratus* SPM, NW_001353090.1; *Takifugu rubripes* Sushi-ichi, AF030881.2; *Autographa californica nucleo polyhedrovirus* Ted, M32662.1; *Schizosaccharomyces pombe* Tf1, M38526.1; Tf2, L10324.1; *Drosophila virilis* Ulysses, X56645.1; *Ceratitis capitata* Yoyo, U60529.1; *Oryzias latipes* LReO-3, BA000027.2; *Sparus aurata* Saugg1, HQ021461.1. Some DIRS1-like elements were also used as phylogenetic outgroup: *Tetraodon nigroviridis* TnDIRS1, AF442732.1; *Tribolium castaneum* TcDIRS1, AY531876.1; *Strongylocentrotus purpuratus* SpDIRS1, biocadmin.otago.ac.nz/fmi/xsl/retrobase/home.xsl.

### Ethics Statement

No specific permits were required for the described field studies. The sampled locations are not privately-owned or protected in any way, and the field studies did not involve endangered or protected species.

## Results

### Characterization of Copia and Gypsy Elements in *R. exoculata*

**Figure 1:**
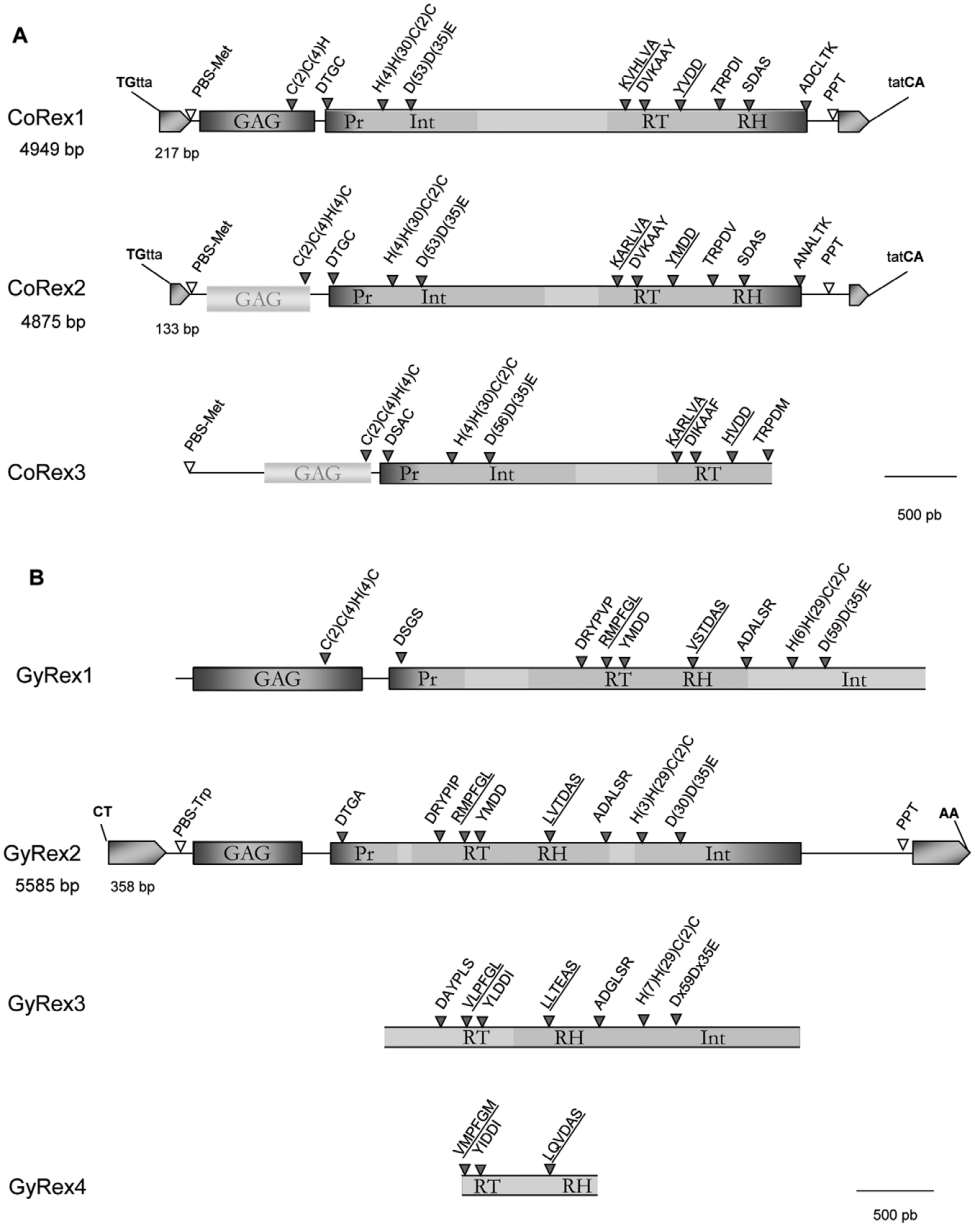
CoRex (A) and GyRex (B) retrotransposons annotation. When an element is described in full-length, its size (in bp), the size of its LTRs and its bordering nucleotides are given. The *gag* and *pol* regions are represented using grey blocks and their conserved domains are indicated by black triangles. Light grey blocks show putative altered *gag* regions. Positions of the Primer Binding Site (PBS) and the PolyPurine Tract (PPT) are indicated by white triangles.

To isolate Copia and Gypsy retrotransposons in the hydrothermal shrimp *R. exoculata*, we performed PCR amplifications using degenerate primers. The CD1 and CD2 primers, designed within the conserved “KARLVA” and “YLDD” motifs of the RT (Figure 1), allowed us to amplify and sequence six Copia fragments of ∼400 bp. The analysis of these fragments revealed 3 families we called CoRex1-3. The GD1 and GD2 primers, designed within the “RMPFGL” and “LTTDAS” conserved motifs of the RT and RH, led to the identification of 4 Gypsy fragments that cluster into 3 families we called GyRex1-3.

A fast and efficient strategy characterizing all parts of a chimeric full-length retrotransposon in 4 to 5 walking steps (Figure S1) was used on the CoRex1-3 and GyRex1-3 fragments. It associates three complementary walking approaches: the ‘PCR walking’ and ‘TE Walking’, as previously described for the characterization of the GalEa and Alvi elements [17], [35], and a new method we developed and called ‘PBS walking’. This method allows the coverage of the region from the Primer Binding Site (PBS) to the RT in only one walking step (see Material/Methods).

CoRex1 is represented by a 4949 bp chimeric consensus sequence (Figure 1-A), which includes two 217 bp LTRs, and is surrounded by the dinucleotides 5'-TG…CA-3′ commonly observed in retrotransposons. The internal region carries a PBS sequence (TGGTAGCAGAGC; position 219), identical to the GalEa1 element PBS and complementary to the 3′ end region of *D. melanogaster* tRNAMet gene, and a putative PolyPurine Tract (PPT) signal (A_3_GA_3_GAG_2_ACGAG; position 4715). CoRex1 comprises two ORFs (Open Reading Frame). The first ORF encodes a *gag* region (288 amino acids) that holds the zinc-finger motif (CX_2_CX_4_HX_4_C) found in all retroviral gag genes. The second ORF exhibits the domains of *pol* region in the order characteristic to Copia: (1) the protease (PR) domain with the typical ‘DSGA’ motif substituted by a ‘DTGC’ motif*;* (2) the integrase (INT) domain with its zinc-finger motif (HX_4_HX_30_CX_2_C) and DD35E signature; (3) the reverse transcriptase and RNaseH (RT/RH) domains containing all the subdomains of RT sequences [11], [12] and the highly conserved TRPDI motif of the RH. CoRex2 is represented by a 4875 bp chimeric consensus sequence (Figure 1-A) harboring shorter LTRs (133 bp) than CoRex1. However, CoRex1 and CoRex2 share the same LTR termini (5'-TGTTA; TATCA-3'). CoRex2 also shares the same PBS as CoRex1 and harbors a putative PPT at the position 4616 (A_2_GAGA_5_G_2_AG_4_GAGA). We identified a 3220 bp *pol* region (our chimeric sequence including a stop codon at the position 1537 and two frameshifts at the positions 1202 and 3934) that exhibits all the Copia domains and signatures. Upstream of its *pol* region, CoRex2 comprises an altered 522 bp sequence that harbors however the *gag* zinc-finger motif and shows similarity with the *gag* region. Finally, we were not able to characterize CoRex3 in full-length. CoRex3 is represented by a 4128 bp chimeric sequence from the PBS (identical to the CoRex1-2 PBS) to the 3′ end of the RT domain (Figure 1-A). All characteristic domains can be found although the *gag* appears highly mutated.

The GyRex1 element is represented by a 4945 bp sequence comprising all domains from the *gag* region to the INT (Figure 1-B). The first 366 amino acid ORF could correspond to the *gag* region, according to similarity searches and the presence of a zinc-finger motif (position 940). The *pol* region (>3330 bp) shows all the signatures from PR to INT domains (but harbors one frameshift). GyRex2 is represented by a 5585 bp chimeric consensus sequence (Figure 1-B), including two 358 bp LTRs surrounded by the dinucleotides 5'-CT…AA-3'. It harbors a PBS sequence (TGGTGACCCTGAAGTA; position 467) complementary to the 3′ end region of a *D. melanogaster* tRNATrp gene and similar to the PBS of the Boudicca element from *Schistosoma mansoni* (AAT98609; E-value = 4e^−157^ between GyRex2 and Boudicca). This allowed us to perform the ‘PBS walking’. A putative PPT signal (A_2_GA_3_T_2_AG_3_AG) is observed at the position 5131. GyRex2 harbors two ORFs: (i) a first 235 codon ORF corresponding possibly to the *gag* region even if no zinc-finger motif can be identified, (ii) a second ORF exhibiting the signatures and domains in the order characteristic of Gypsy *pol* region. GyRex3 is only represented by a fragment of the *pol* region (2698 bp) that includes the RT, RH and INT domains (Figure 1-B).

The CoRex1-3 and GyRex1-3 characterization led also to the artifactual amplification of 3 new non-targeted Gypsy elements (GyRex4-6). GyRex4 was identified in its RT/RH domains (Figure 1-B) and appears highly divergent from GyRex1-3 (<33% identity on the 898 bp). GyRex5 is characterized by a 671 bp INT sequence that encompasses a zinc-finger and the DD35E signature. Interestingly, both GyRex4 and GyRex5 show high similarity to an element from the gilt-head bream *Sparus aurata* we called Saugg1 (HQ021461.1), which possesses the same structure than Gmr1-like retrotransposons. Gmr1-like elements are unconventional Gypsy retrotransposons in which the INT domain lies upstream, rather than downstream, of the RT domain [59]. Since the GyRex4-5 sequences do not overlap themselves, they could thus possibly belong to the same element. GyRex6 is represented by a 1160 bp sequence from the PBS position to the beginning of the *pol* region (‘DTGA’ motif of PR domain at the position 1145), and includes a potential 221 codon *gag* ORF. GyRex6 differs from GyRex1 and GyRex2, but here again we cannot exclude that it does not correspond to a portion of GyRex3-5 because of the lack of overlapping sequences.

Sequences corresponding to three other transposable elements were also identified: two new LINE retrotransposons (LiRex1-2) and one transposon (T-Rex1). LiRex1 (354 bp) appears highly corrupted, although the RT4 motif of the reverse transcriptase [11] is still detectable. LiRex2 (563 bp) is more conserved with the recognizable RT5, RT6 and RT7 motifs. Finally, the T-Rex1 sequence (675 bp) shows high similarity with a transposon from the sea urchin *Stongylocentrotus purpuratus* (XP001188275.1, E-value = 6e^−54^).

The *R. exoculata* specimens were collected on hydrothermal vents where they could have been subjected to stresses due to the hypervariability of the environment. They were also exposed to many stresses related to fishing conditions (decompression, temperature variations…) that could also favor the activation of TEs. We performed RT-PCRs on the *R. exoculata* transcripts using primers specific to each element. Transcriptional activity was revealed for CoRex1 and CoRex2. Three CoRex1 (>97% identity) and five CoRex2 (>87% identity) transcript sequences were identified (Table S1), highlighting a preponderance of CoRex2 on the other Copia families within *R. exoculata*. No transcript could be detected for GyRex1-4 and Corex3, which however do not attest to their inactivity in other specimens or conditions.

To determine the CoRex1-3 and GyRex1-6 distributions among species related to *R. exoculata*, we PCR-screened 4 other Alvinocarididae species (*A. lusca*, *A. markensis*, *C. chacei* and *M. fortunata*) as well as two closely related non-hydrothermal shrimps (*C. crangon* and *P. serratus*; [60]) using few combinations of specific primers for each element (Table S1). Elements related to CoRex1-3 and GyRex2 are detected in all hydrothermal shrimps, except CoRex1 that could not be identified in *M. fortunata*. This led to the identification of several new elements: CoAlma1 (*A. markensis*) and CoAllu1 (*A. lusca*) from the CoRex1 family (>97% identity); CoMiro2 (*M. fortunata*), CoAlma2 and CoAllu2 from the CoRex2 family (>87% identity); CoAlma3 and CoMiro3 from the CoRex3 family (>90% identity); and GyMiro2 and GyAlma2 from the GyRex2 family (>79% identity). Finally, Gychoro2, an element that belongs to the same family than GyRex4 (93% identity), was detected in *C. chacei,* whereas GyRex1, GyRex3, GyRex5 and GyRex6 could not be detected in any other species.

### Copia and Gypsy Retrotransposons in Crustaceans

**Figure 2:**
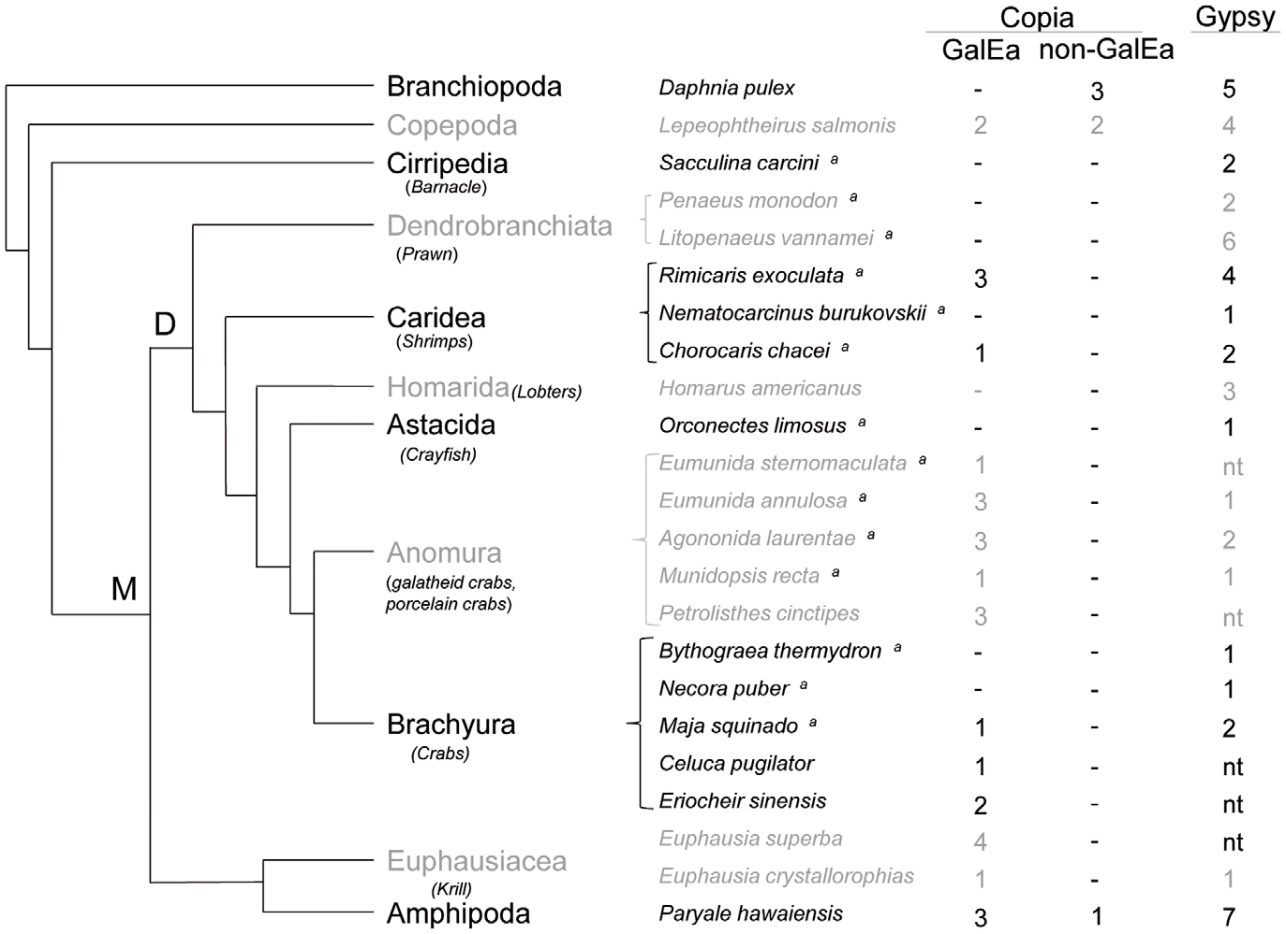
Number of Copia and Gypsy elements studied in crustaceans. Genetic relationships between crustacean classes and orders are represented by a tree topology reconstructed from previous studies (Regier *et al.* 2010, Giribet and Edgecombe, 2011; Ahyong and O’Meally, 2004). **M**: Malacostraca, **D**: Decapoda. For Copia retrotransposons, GalEa and non-GalEa elements are distinguished. Only a few representatives of the Copia elements described *in D. pulex* were studied. nt: not tested; -: no element detected; ^a^ species screened using degenerate PCRs.

To estimate the diversity of Copia and Gypsy elements within crustaceans, we PCR-screened 25 decapods and crustacean species using degenerate primers. We additionally looked for retrotransposons in the crustacean genomic and transcriptomic databases using similarity searches. These two complementary approaches led to the identification of 35 Copia and 46 Gypsy elements distributed among 15 and 18 species, respectively (Figure 2). Sixteen and twenty-nine of these Copia and Gypsy elements were included in phylogenetic analyses based on the RT/RH domain and the remaining sequences were classified using a BLAST-based approach (see Materials and Methods and Table S2).

**Figure 3:**
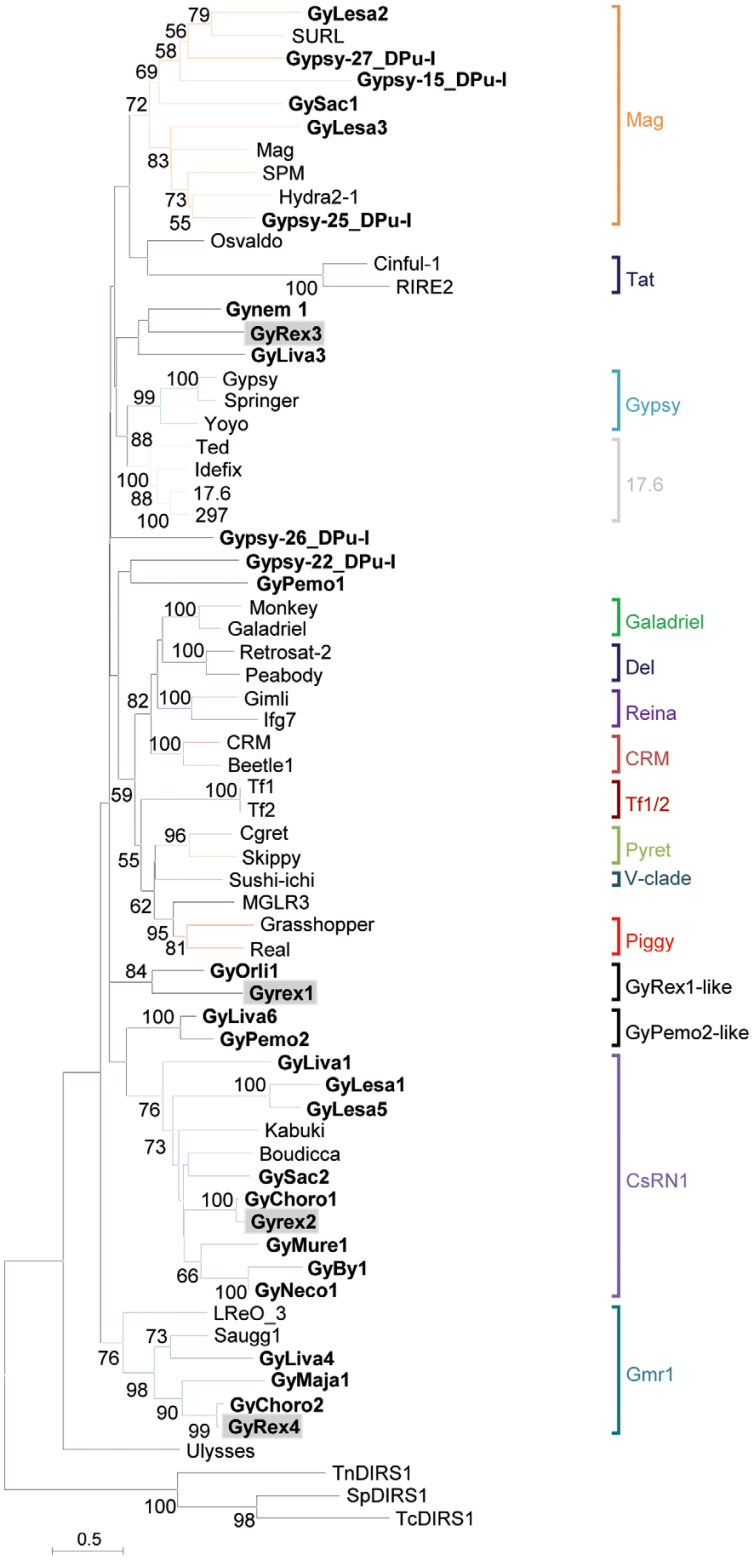
Phylogenetic relationships among Gypsy retrotransposons inferred from Neighbor-Joining analysis of RT/RH amino acid sequences. The crustacean elements are indicated in bold and the four *R. exoculata* elements (GyRex) are highlighted in grey. Statistical support (>50%) comes from non parametric bootstrapping using 100 replicates. DIRS1-like sequences were used as outgroup.

Gypsy retrotransposons from crustaceans are divided in several clades (Figure 3). One third of the elements group in the CsRN1 clade, including elements from the copepod salmon lice *Lepeophteirus salmonis* (GyLesa1 and GyLesa5), the cirriped barnacle *S. carcini* (GySac2) and diverse decapods such as *R. exoculata* (GyRex2), crabs (e.g. GyBy1 from *B. thermydron*), squat lobsters (GyMur1 from *M. recta*). This clade also includes the GyPaha1-3 elements from the amphipod *P. hawaiensis* (Table S2). The Mag clade encompasses seven elements from the branchiopod *D. pulex* (GyDpu15 and GyDpu25), the copepod (GyLesa2 and 3), the cirriped (GySac1), and the krill *E. crystallorophias* (GyEcrys1). To date no Mag clade element has been identified in decapods. Four elements appear to be related to the Gmr1 clade: GyRex4 and Gychoro2 (hydrothermal shrimps), GyMaja1 (spiny spider crab *M. squinado*) and GyLiva4 (prawn *L. vannamei*), which are the first Gmr1-like elements described in protostomes. Several new clades may be also identified using the crustacean elements. For example, GyRex1 seems closely related to GyOrli1 (crayfish *O. limosus*), and the GyLiva6 and GyPemo2 elements from the prawns *L. vanameii* and *P. monodon* are grouped in a very well supported clade. The remaining elements appear to be more or less dispersed within the phylogeny and do not belong to any previously identified clade. Finally, the Gypsy tree mostly differs from the crustacean phylogeny. Clades include elements from distant species and elements from one species belong to distant clades. For example, in *R. exoculata*, GyRex2 is a CsRn1-like element and GyRex4 a Gmr1-like, while GyRex1 and 3 do not belong to any previously defined clade. Three elements from *D. pulex* group into the Mag clade while the two others remain isolated in the phylogeny. The four GyLiva (*L. vannamei)* are divided among four different clades, and the GyLesa (*L. salmonis*) and GySac (*S. carcini*) elements are split among the CsRN1 and the Mag clades.

**Figure 4:**
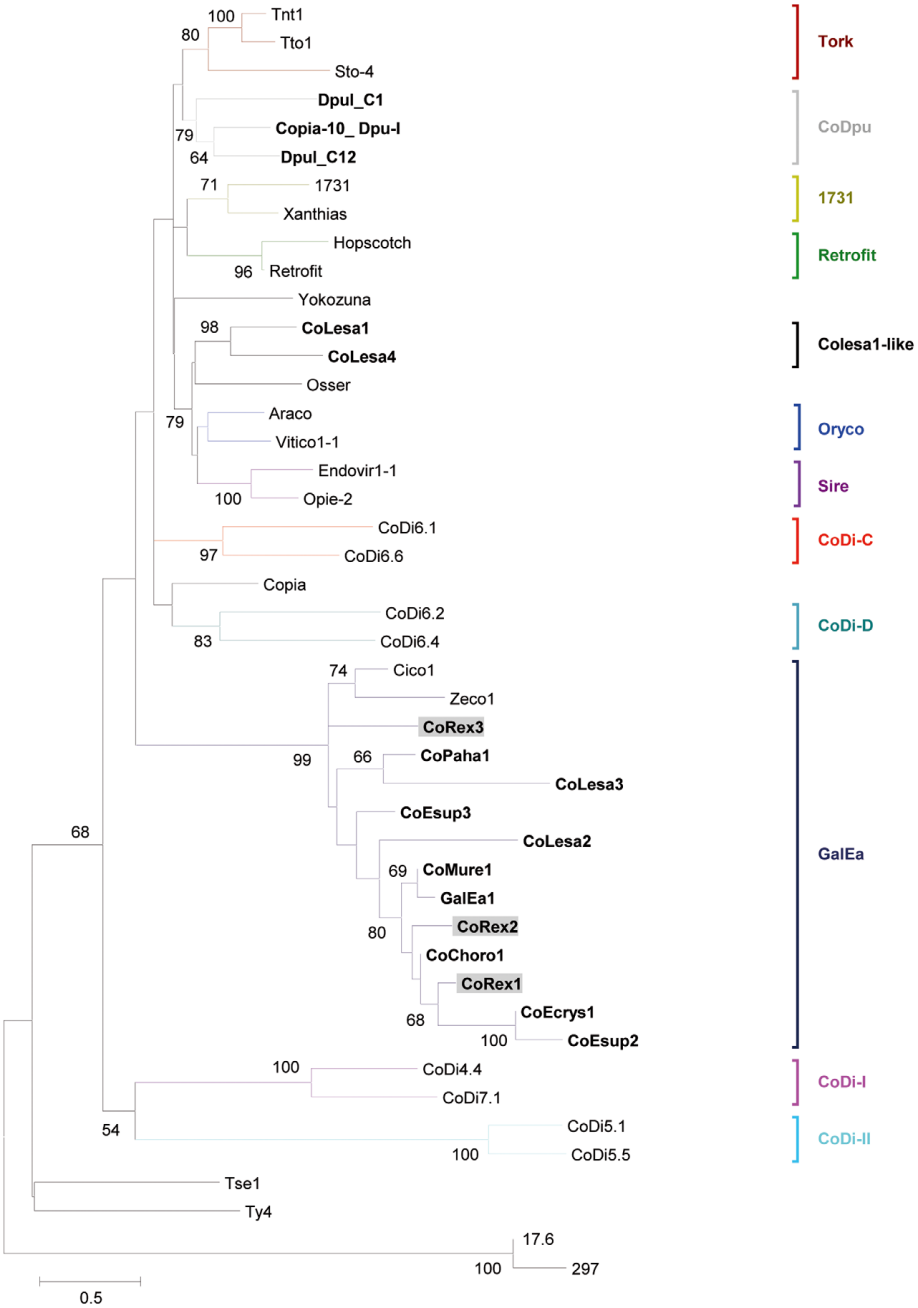
Phylogenetic relationships among Copia retrotransposons inferred from Neighbor-Joining analysis of RT/RH amino acid sequences. The crustacean elements are indicated in bold and the three *R. exoculata* elements (CoRex) are highlighted in grey. Statistical support (>50%) comes from non parametric bootstrapping using 100 replicates. Gypsy sequences were used as outgroup.

In contrast to the Gypsy retrotransposons, the 35 Copia elements from crustaceans appear much less diversified, as they all fall into three clades (Figure 4). Seven of these sequences were previously described as GalEa-like elements [35], including the well-annotated GalEa1 elements (galatheid squat lobsters). Twenty-one new elements, including the CoRex1-3 retrotransposons, belong to this highly supported GalEa clade (Figure 4 and Table S2). It is interesting to note that in terms of diversity various species harbor several GalEa-like families (e.g. at least 4 detected in the *E. superba* transcriptome, 3 in *P. hawaiensis* and 3 in *E. annulosa* genome). The 6 remaining elements belong to three different clades: (i) The three elements from *D. pulex,* which correspond to the two subgroups defined by Rho *et al.* [32], grouped together in a single clade we called CoDpu; (ii) CoLesa1 (ADND02013164.1) and Colesa4 (ADND02043341.1) from the copepod *L. salmonis* grouped in a new clade we called CoLesa1-like that is related to the Sireviruses; and (iii) similarity searches on the CoPaha4 element from the amphipod *P. hawaiensis* revealed that this element is likely related to the Hydra clade (Hydra1-2, E-value = 9e^−50^). Interestingly, an additional screen of another Daphnia species, *Daphnia pulicaria* (http://wfleabase.org/blast/), could only reveal Copia elements that belong to the CoDpu clade (data not shown).

## Discussion

### Crustaceans: a Suitable Study System for Transposable Element Dynamics

Given their abundance, high level of phylogenetic diversification, huge diversity of environment and life styles, and extended range in C-values with particularly large genomes (460-fold variation from 0.14 to 64.62 pg [22]), crustaceans appear a worthy focus for comparative study of metazoan genomes at an intermediate scale (*i.e.* within a subphylum or a class). Crustaceans also appear as one suitable system for a comparative genome evolution study with Hexapoda, one of the most studied group in biology. Indeed, crustaceans are, for example, the second most studied group of “invertebrates”, after hexapods, for genome size reports (318 species reported in the Genome Size Database, Gregory 2008). However, crustaceans remain greatly underrepresented in genomics. Only few large-scale genomic sequencing analyses, restricted to branchiopods, have been performed [32]. Nevertheless, the emergence of next-generation sequencing technologies now allows comparative genomic studies for non-model species and/or large genomes [61]–[64], and led to the recent acquisition of genomic and more especially transcriptomic data for several crustacean species.

Among crustaceans, we focused on *R. exoculata,* listed as a model organism of an extreme deep-sea environment (CAREX, 2010), which dominates the vagile megafauna at many hydrothermal vent sites along the Mid-Atlantic Ridge. *R. exoculata* has been studied in many aspects, such as biogeography/population genetics [65], [66], bacterial symbiosis association [41], [67] and response to stress [68], [69]. *R. exoculata* could also represent an interesting model species for transposable element dynamics because of its extremely variable environment. Our present study, combined with the previous analysis of DIRS1-like retrotransposons in decapods [17], allows us to describe a great diversity of transposable elements in this species. At least 13 TE families are identified, including 2 tyrosine recombinase encoding elements (Alvi1-2), 3 Copia (CoRex1-3) and 6 Gypsy (GyRex1-6) LTR-retrotransposons, as well as 2 LINEs (LiRex1-2) and one transposon (T-Rex1). We noticed that element detection using the degenerate primers approach is usually fairly easy in this species, which confirms the tendency observed during the detection of DIRS1-like elements in hydrothermal shrimps. This seems to be also the case for galatheid squat lobsters (e.g. *E. annulosa*), where a large diversity of retrotransposons is described (DIRS1-like, [17]; GalEa-like, [35]; Gypsy and Pao/Bel, [36]). We hypothesized [17] that such results can be partly related to the copy number in such species having a large genome size [23].

### Copia Retrotransposons Seem Relatively Rare in Crustaceans

Thirty-four Copia retrotransposons are now identified in crustaceans. However, we often observed a lack of detection or very low PCR signals in the species we screened for Copia elements using degenerate primers. Although the degenerate primers were designed within very well conserved motifs (Table S3) and are known to be efficient [35], [47], [70], PCR-screenings led to the identification of 9 Copia retrotransposons in only 7 of the 14 species tested, including 6 Caridea and Anomoura spp. Besides, an additional PCR-screening of 10 other diverse crustaceans could not lead to the detection of any Copia elements. Set apart the choice of primers, the lack of PCR signal could simply be due to the rarity of the elements or their absence from the species studied. Indeed, even if CoDpu elements seem relatively abundant in *D. pulex* [32], the absence or rarity of Copia elements could be a genomic feature frequent in crustacean species. For example, none of these retrotransposons have been reported in repetitive element families of *P. monodon* [71]. Likewise, we could not identify any Copia elements in the well-sequenced transcriptome of *L. vannamei* (141030 contigs available in the *Penaeus* Genome Database: http://sysbio.iis.sinica.edu.tw/page/).

This feature is however not restricted to crustacean species since LTR-retrotransposons are known to be less abundant in animals [10]. Compared to their close relatives, the crustaceans do not differ from the other species. Indeed, de la Chaux and Wagner [21] recently reported that Copia elements have a small relative abundance in hexapods, Copia elements being usually much rarer than the Gypsy or Pao/Bel retrotransposons. They even appear to be absent in one species, *Ixodes scapularis*. In general, it has been shown that Copia elements constitute only a small proportion of LTR-retrotransposons identified in numerous metazoan genomes [21], as well as in fungi [72]. For example, only few were detected in the comparative analysis of TEs content from salamanders [73] and none are described in the draft genome of the pearl oyster [74].

### Copia and Gypsy Retrotransposons: Two Opposite Dynamics in Crustaceans

In addition to the fact that Copia elements are much scarcer than Gypsy in metazoan genomes, Copia elements appear clearly less diverse. While studying the evolutionary history of LTR-retrotransposons in eukaryotes, Llorens [20] observed that Gypsy elements have been more successful than their Copia counterparts during evolution. The authors hypothesized that the higher phenotypic plasticity of Gypsy retrotransposons allowed them to diversify much more than Copia elements at distinct geological eras. Our phylogenic analyses of crustacean LTR-retrotransposons also fit this observation. We observed two diametrically opposed patterns for crustacean Copia and Gypsy elements (Figure 3 and 4). Even within a single species such as *R. exoculata,* its GyRex and CoRex elements follow this pattern. The Gypsy elements appear very diverse, widely dispersed among the phylogeny and many clades of Gypsy are represented or are newly described. This large diversity of Gypsy retrotransposons is probably inherited from an ancestral polymorphism in crustacean lineage, where several active element copies within species have been maintained. For example, many crustacean elements belong to the Mag clade, which is believed to be one of the oldest Gypsy clades [20]. The newly described clades (Gyrex1-like, Gynemo2-like; Figure 4) could also result from a diversification of Gypsy elements during the evolution of crustaceans. Alternatively, a higher rate of horizontal transfers could also lead to such diversity, but to date no argument supports this hypothesis. In contrast, the diversity of Copia retrotransposons in crustaceans appears much more restricted and related to the host species. The GalEa clade appears highly predominant comprising 29 elements detected in Decapoda, and more generally in Malacostraca (Figure 2). Only two elements from the copepod *L. salmonis* group into the new CoLesa1 clade, and one element from the amphipod *P. hawaiensis* appears to belong to the Hydra clade. Finally, all the Daphnia elements form the CoDpu clade.

The dynamics of transposable elements is a complex concept, which combines numerous aspects such transposition control mechanisms by the elements themselves and/or the host genome, the element activation by environmental changes (at the genome or ecological levels), the mutation rate, the host migration, the possible domestication events, etc. Moreover, many of these parameters are subject to random events (drift). To get a mental picture of GalEa dynamics, and presumably those of some other elements, we can draw an analogy with a “domino days spreading” branching process in which successive amplifications may interact positively. During the famous worldwide event of toppling domino stones, we can follow the propagation of domino falls along various branches and through several major figures that encompass large, but variable numbers of dominoes. Elements could be represented by the dominoes and the number of copies by the number of falling stones, helping to visualize the TE diffusion within taxa and species during evolution (except that domino structures are pre-designed). Like domino bricks following a restricted number of lines before toppling large structures, few active TEs copies must be inherited prior to a transposition “burst”. Many factors could lead to such expansion within a species. For example, it is well illustrated that TE transposition can be activated by stresses [7], [9], [75] or the colonization of a new environment [76]. It has also been hypothesized that variations in the TE repertoire could promote or be associated with the emergence of new lineages, species, populations or subpopulations [77]–[79]. Later on, the large domino structures allow the progression to the next structure *via* several paths. Similarly, an initial amplification increases the proportion of young active elements, which allow subsequent derived amplifications in some random lineages, possibly through the transposition of few master copies. Furthermore, the limited number of toppling dominoes between figures may facilitate the random breaking off of their progression along some paths. Similarly, evolutionary forces may drive the extinction of some elements within a lineage when elements are maintained too long at a low copy number. In a funny parallel, the high diversity of dominoes features may also reflect the element diversity and the evolution. Changes in the material or color of dominoes, which are much more numerous in the figures, may reflect TEs mutations and the recent use of “slow stones” may represent variable speed of evolution. Likewise, to ensure the success of major figures, builders design rescue paths in case of failure of the main circuit, which can easily be compared to the TE dynamics through horizontal transfers.

In the case of the crustacean phylogeny, such a model could have led to the current GalEa distribution and could explain the three transitions observed in the Copia content: (i) the expansion of GalEa-like elements in a common ancestor; (ii) the predominance of GalEa-like elements in decapods and euphausiaceans; and (iii) the loss of Copia elements in some species. The expansion of GalEa-like elements prior to multicrustacean radiation is supported by their presence in most Malacostraca and in the only copepod tested. It could be hypothesized that GalEa-like elements have been horizontally transferred to the multicrustacean ancestor (*i.e.* Copepoda, Cirripedia and Malacostraca according to Regier [80]) and then invaded its genome. However, since they are present in various metazoans (see section below), GalEa-like elements should have been already present in the multicrustacean common ancestor. The GalEa-like element absence in branchiopods remains to be confirmed by the study of other species outside the daphnia group. The phylogenetic distance between Branchiopoda and Multicrustacea supports this hypothesis. Indeed, the relationships within Pancrustacea remain controversial as several studies describe Branchiopoda as a sister-group to Hexapoda instead of Multicrustacea [81]–[83]. In such a case, Copia retrotransposons from branchiopods are expected to be as different from GalEa as those observed in hexapods [20].

In addition to the GalEa-like element distribution, the detection of several other Copia elements in amphipods and copepods suggests that the Copia repertoire of crustacean or multicrustacean ancestor comprises elements from several clades. Since the GalEa-like elements appear to be exclusive to decapods and euphausiaceans, by implication the other Copia retrotransposons have been rarely amplified and have been progressively lost. Most likely, a slow mutational decay of other Copia retrotransposons, which are usually in low copy number except in plants [21], [35], [72], led to this loss in many lineages. Besides, the success in maintaining GalEa-like elements within multicrustaceans appears to be species- or lineage-specific. The fact that only some Copia are able to counteract the evolutionary erosion forces suggests that the dynamics of the different elements may be related to particular ability of each of them to amplify under peculiar conditions in some genomes. For example, the tobacco Tnt1 retrotransposons tightly control their activation by restricting expression to specific conditions, as they possess in their promoter regulatory motifs similar to those involved in activation of plant defense genes [7], [75], [84]. GalEa-like elements seem to have been lost in few species, such as prawns, while they seem to have undergone some secondary expansions in others infraorders, such as in galatheid squat lobsters or caridean shrimps. This could explain their uneven distribution among Decapoda. Interestingly, similar expansions of DIRS1-like elements have also been observed in these lineages [17].

To reinforce the idea that few specific Copia elements could, from time to time, increase their transpositional activity and so broaden their occurrence in some particular host taxa, it appears necessary to study Copia diversity in other metazoan groups at roughly the same scale of study. For this, it may be interesting to survey the distribution of CoDpu-like elements among Branchiopoda, and/or to study Copia elements diversity in another taxon such as Hexapoda. To date, six clades of Copia retrotransposons have been described in wingled hexapods: 1731, Copia, GalEa, Humnum, Mtanga and Tricopia [20]. Interestingly, as observed in crustaceans, the distribution of TE clades among species appears also highly related to the host phylogeny. For example, whereas the Copia clade is widely distributed in Insecta [85]–[87], the Tricopia, Mtanga and Humnum clades have been detected in only one species [20], [88].

### GalEa-like Retrotransposons in Eukaryotes

The success of GalEa-like elements in crustaceans raises the question of their distribution in others organisms. When they defined the GalEa clade, Terrat *et al*. [35] described GalEa1 related elements in 3 fishes and 1 acidian. The GalEa clade is actually more widely distributed among animals. We retrieved GalEa-like retrotransposons through BLAST searches using GalEa1 and Zeco1 *pol* domain as queries, which now allow us to report such elements in more than 50 species (Table S2). Many of these species are of course crustaceans (16 species). There are also numerous fishes (18 species), as GalEa-like elements appears widely distributed in teleost fishes, which are the subject of many sequencing projects. GalEa-like elements are also present in diverse molluscs (7 species), as well as some Chordata, Cnidaria, Ctenophora, Echinoderma and Hemichordata. Two elements (CoPorcru1 and CoPorcru2) were also detected outside metazoans, in the red algae *Porphyridium cruentum*. This fits the previous identification of some similar GalEa-like elements in another red algae, *Porphyra yezoensis* (PyRE10G, AB286055) and suggests that GalEa-like elements are probably ancient in eukaryotes, at the exception of the hypothesis of multiple horizontal transfers. To determine the relatedness between these different GalEa-like retrotransposons, we performed a phylogenetic analysis based on the RT-RH domain of 42 elements that represent 33 species (Figure S2). Within the well-supported GalEa clade (bootstrap value 92%), the two red algae elements (CoPorcru1-like) form a distinct group from all other elements. Three other groups can also be defined. Almost all elements from crustaceans group in a same subclade (CoRex1-like), except CoRex3 and CoLesa2. Likewise, all elements from fishes belong to a monophyletic group (bootstrap value 97%) and form, with CoCre1 (*Crepidula fornicata*) and CoSaccoglo1 (*S. kowalevskii*), a subclade we called Zeco1-like (bootstrap value 89%). The last subclade, CoPali1-like (bootstrap value 99%), contains one element from the sea urchin *Paracentrotus lividus* and one from the cuttlefish *Sepia officinalis*. The remaining elements, especially those from molluscs, appear more or less dispersed within the phylogeny. GalEa-like elements have a widespread distribution, being highly represented in at least 3 groups of organisms: Malacostraca, Teleostei and probably part of Mollusca. For a better understanding of the distribution of GalEa-like retrotransposons, we wonder whether their predominance is a peculiar feature of Malacostraca, or whether similar feature can be observed in other species clades.

## Supporting Information

Figure S1 **Characterization strategy of full-length LTR-retrotransposons.** A copia retrotransposon is used as example. For each of the five steps, the known part of the element is represented by a full line whereas the walking part is indicated by colored dotted arrow: red, PCR or TE Walking; green, PBS Walking; purple: PCR using specific primers. The conserved domains used to design the degenerate primers and the PBS sequences are represented by blue and green triangles, respectively.
(TIF)

Figure S2 **Phylogenetic relationships among GalEa-like retrotransposons inferred from Neighbor-Joining analysis of RT/RH amino acid sequences.** Statistical support (>50%) comes from non parametric bootstrapping using 100 replicates. Two to three representative elements of the other Copia clades are also included to the phylogeny. Gypsy sequences were used as outgroup.
(TIF)

Table S1 **Report of the sequences obtained from PCR approaches.** For each element, the host species, name, length and accession number are given, as well as the PCR methodology and primers used.
(XLSX)

Table S2 **List of GalEa-like retrotransposons identified.** For each element, the corresponding host species and the accession number(s) are indicated. The GalEa nature of the elements was determined following different classification methods: Figure B and SupData E correspond to the phylogenetic analyses; BlastP to the BLAST-based classification method, for which the best GalEa and non-GalEa hits are given with the corresponding E-values.
(XLSX)

Table S3 **Comparison of CD1 and CD2 primers with Copia sequences.** Dissimilarities at nucleic or amino-acid levels are indicated in red.
(XLSX)
