# Supplementary figures and images for: LTR-Retrotransposons in R. exoculata and Other Crustaceans: The Outstanding Success of GalEa-Like Copia Elements
Source: PLoS One. 2013 Mar 4;8(3):e57675. doi: 10.1371/journal.pone.0057675 (PMC3587641; doi:10.1371/journal.pone.0057675)

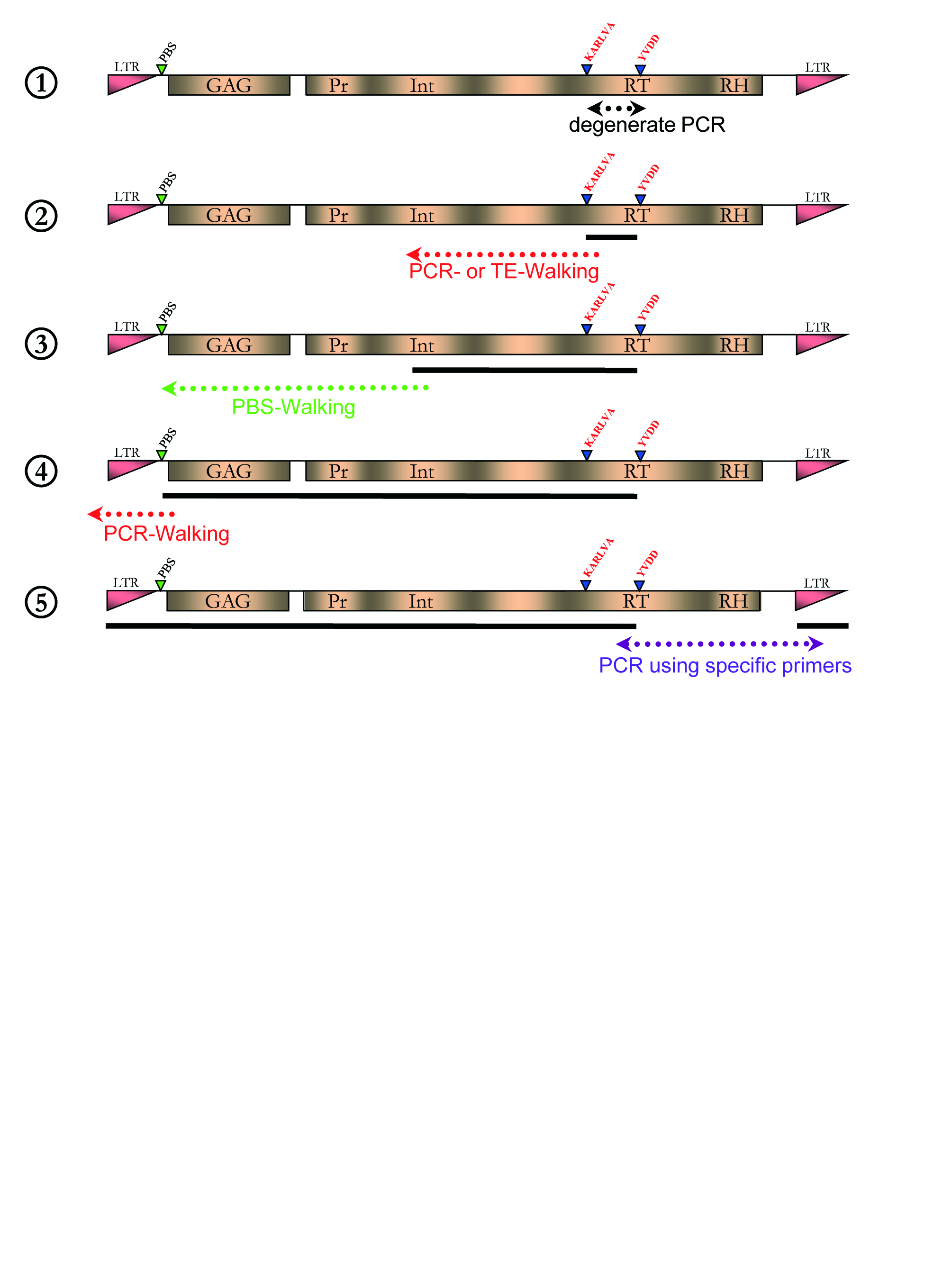

Supplement: Figure S1 — Characterization strategy of full-length LTR-retrotransposons. A copia retrotransposon is used as example. For each of the five steps, the known part of the element is represented by a full line whereas the walking part is indicated by colored dotted arrow: red, PCR or TE Walking; green, PBS Walking; purple: PCR using specific primers. The conserved domains used to design the degenerate primers and the PBS sequences are represented by blue and green triangles, respectively. (TIF) [file pone.0057675.s001.tif]

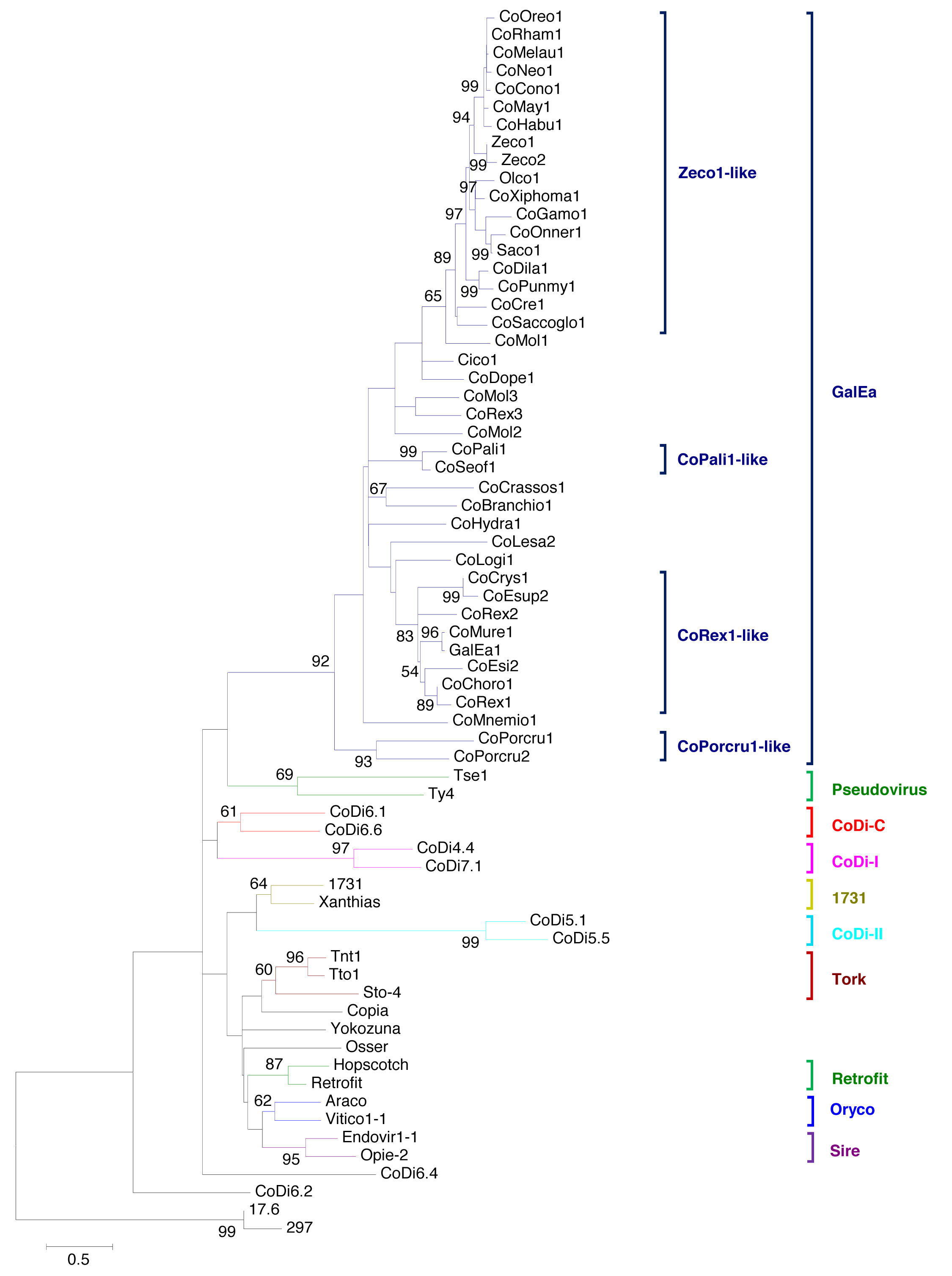

Supplement: Figure S2 — Phylogenetic relationships among GalEa-like retrotransposons inferred from Neighbor-Joining analysis of RT/RH amino acid sequences. Statistical support (>50%) comes from non parametric bootstrapping using 100 replicates. Two to three representative elements of the other Copia clades are also included to the phylogeny. Gypsy sequences were used as outgroup. (TIF) [file pone.0057675.s002.tif]
